# Analysis of the Horizontal Distribution of Sampling Points for Gas Concentrations Monitoring in an Open-Sided Dairy Barn

**DOI:** 10.3390/ani12233258

**Published:** 2022-11-23

**Authors:** Provvidenza Rita D’Urso, Claudia Arcidiacono, Giovanni Cascone

**Affiliations:** Department of Agriculture, Food and Environment (Di3A)—Building and Land Engineering Section, University of Catania, Via Santa Sofia n° 100, 95123 Catania, Italy

**Keywords:** sampling locations, sampling strategies, number of repetitions, gas concentrations, variability, gas distribution

## Abstract

**Simple Summary:**

The livestock sector is considered a source of negative impacts on the environment. The measurement of gas concentrations inside dairy barns has a relevant role in the implementation of reduction strategies as well as in improving the indoor air quality for both workers and animals in the barn. The aim of this research study was to contribute to assessing the methodological choices in measurement campaigns of pollutant gases in open dairy barns. Based on the assessment of data variability, the location and the number of sampling points influenced the measurement of the gas in an open dairy barn.

**Abstract:**

In the livestock sector, the monitoring of gas concentrations plays a relevant role in the implementation of mitigation strategies, as well as the improvement in the indoor air quality for both workers and animals in barns. In the present study, measurements of gas concentrations (NH_3_, CH_4_, and CO_2_) at different locations were carried out in an open dairy barn using a commercial photo-acoustic spectroscopy system. Measurement variability for different datasets was computed to contribute to the evaluation of the sampling strategy in the measurement campaign. The results showed that the position of sampling locations (SLs) significantly influenced (*p* < 0.001) the variability in the gas distribution. Specifically, the SLs located on the perimeter of the building had the highest variability. The number of SLs utilised for determining the mean value of gas concentration in the barn had a significant influence on NH_3_ (*p* < 0.001), CO_2_ (*p* < 0.001), and CH_4_ (*p* < 0.001) in both the central zone and the perimeter one. At least three SLs were necessary to obtain a mean value of gas concentration that reduced the variability to under the 10% in the central zone and 16% in the perimeter one. Moreover, the NH_3_ concentrations obtained as the mean value of the gas measurements at two SLs with a 10 m distance had a lower variability compared to those recorded at two SLs located at 5 m.

## 1. Introduction

The livestock sector impacts the air through the release of ammonia (NH_3_) and greenhouse gases (GHG), such as methane (CH_4_) and carbon dioxide (CO_2_) [1,2]. The monitoring of these pollutants represents a relevant activity for the definition of new measures or the implementation of current mitigation strategies to reduce the impacts on the environment from the livestock sector [3]. Among the main impacts on the environment, GHG release is responsible for global warming, whereas NH_3_ is responsible for eutrophication, acidification, and particulate matter formation [4]. Besides the impacts on the environment, high concentrations of NH_3_ negatively affect animal health [5].

In the European context, dairy farms are mainly naturally ventilated buildings characterised by the presence of wide openings to reduce heat stress on animals, especially during warm periods [6,7,8,9].

In the most recent literature studies, many investigations have been carried out to identify proper measurement strategies to acquire data on gas concentrations. Since in many studies it was found that the gas distribution in the barn is not uniform [10,11,12], the planning of the sampling strategies requires a crucial choice regarding the selection of representative sampling locations (SLs). Moreover, obtaining reliable measurements is carried out by the sequence of the measurement and is performed by the instrument, as described by Rom and Zhang [13]. In detail, they recommended avoiding rapid changes between high and low concentration levels. Reducing biases in the acquisition of data can improve the barn management as well as the estimation of emissions, which is mainly based on an indirect balance method for these barn typologies. In previous studies, D’Urso et al. [12] found that the highest gas concentrations were in the central area of the barn along the feeding alley. Janke et al. [9] assessed five sampling strategies in a naturally ventilated dairy barn in northern Germany and found that the sampling strategy could influence the underestimation and overestimation of gas emissions. Although long-term measurements with a single SL may provide satisfactory outcomes [8], the results for a shorter duration of measurements could have more uncertainties in the localisation of SLs [14]. In fact, the analyses performed by König et al. [14] in a naturally ventilated dairy barn revealed that the sampling duration and the number and location of indoor SLs affected the uncertainty of the ventilation rate. Moreover, they found an increased uncertainty up to 15% by using only one SL. For naturally ventilated buildings, the recent VERA test protocol [15] recommended placing sampling locations in the middle of the barn for symmetrical structures and at a distance of 2 m from the walls for open barns. Moreover, it was recommended that there be at least one SL per ten meter length of the barn. However, no evidence was found in the literature on the adequate distance and number of SLs.

Furthermore, the main studies in this field have focused on sampling strategies in typical buildings in Northern Europe [9,14]. In these studies, the structures have openings in the walls to provide ventilation, especially in a warm climate. These openings are often equipped with curtains, which are usually utilised in winter. On the contrary, in the Mediterranean area, the dairy building is a typical open structure characterised by the absence of perimeter walls [12]. Moreover, the natural ventilation system is combined with the use of fans and sprinkler systems, which are mainly switched on during warm periods to reduce heat stress for the animals [16,17]. According to the literature, the computation of the average gas concentration in the barn is obtained by computing the mean value of the gas concentration acquired at different SLs in the barn [18,19,20]. Since the factors that influence the gas concentration values in the barn are related to barn typology and management and environmental conditions [12,21], it is of interest to quantify the variability in the gas concentration values in relation to the number and distribution of the SLs in the barn. In fact, data variability due to the number and distribution of SLs has not yet been investigated.

Therefore, this study aims at filling the above-mentioned gaps by studying the number and distribution of SLs in an open barn located in the Mediterranean area. In detail, the objectives were as follows: (1) analysing the standard deviation of gas concentrations at different locations in the barn; (2) comparing the variability of gas concentration in the barn by using two or three SLs; (3) assessing the variability of gas concentrations when an SL is located five or ten distance from the other SLs.

## 2. Materials and Methods

### 2.1. Experimental Barn and Period of Investigation

**Figure 1:**
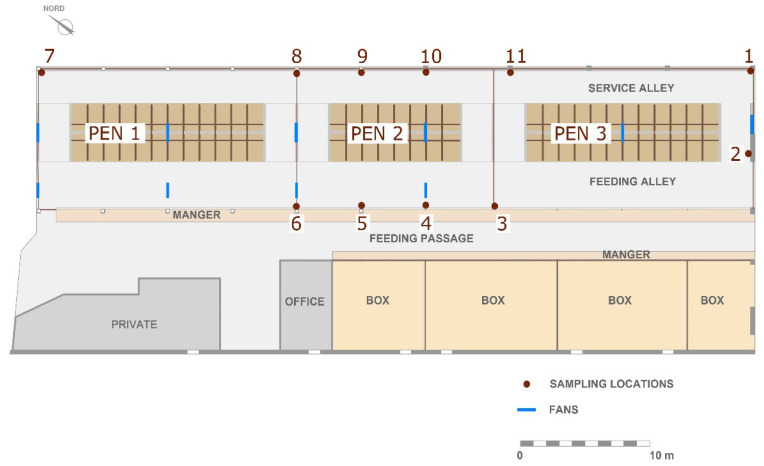
Plan view of the barn under study with the position of the sampling locations and fans.

The collection of measurements was carried out in a cubicle free-stall dairy barn located in the province of Ragusa (Italy) from 21 May to 1 June 2018. During the period of investigation, the indoor and outdoor air temperature was 20.76 ± 5.3 °C and 19.45 ± 4.8 °C, respectively, and indoor and outdoor air velocity was 0.82 ± 0.51 m s^−1^ and 1.44 ± 0.79 m s^−1^. The structure was 55.50 m long and 20.80 m wide. In the barn, the SE, NE, and NW sides were completely open, whereas the SW side was closed by a continuous wall with small openings. The symmetric roof has a central ridge vent with a ridge height of 7 m and an eave height of 4 m. The barn was equipped with a cooling system, composed of a fogging system with fans in the resting area and a sprinkler system with fans in the feeding alley. The 64 head-to-head cubicles were organised in three pens on a concrete floor (Figure 1). The cubicles were arranged in two rows with concrete kerbs filled with sand. On the other side of the feeding lane, different boxes with deep litter were located in the barn.

### 2.2. Measurement of Gas Concentration

Measurements of NH_3_, CH_4,_ and CO_2_ were carried out at eleven SLs using an infrared photo-acoustic spectroscope (INNOVA) composed of a Multigas Monitor mod 1412i and a multipoint sampler 1409/12 (Lumasense Technology A/S, Ballerup, Denmark). The sampler system was made of AISI-316 stainless steel and PTFE (polytetrafluoroethylene) tubing to minimise the adsorption of samples. The system had 11 inlet channels. An air-filter was attached to the end of each tube to keep the sampler free of particles. The eleven SLs, shown in Figure 1, were located 20 cm from the barn floor in the animal-occupied zone (AOZ) according to the findings of Arcidiacono et al. [22]. In the analyses, the SLs were subdivided into three areas based on the localisation within the barn: central SLs (SL03-SL04-SL05-SL06); perimeter SLs (SL08-SL09-SL10-SL11); and corner SLs (SL01-SL02-SL07). The central and perimeter SLs were located in PEN2 in order to study different repetitions in space in the same pen. The distance between the two SLs of the central and perimeter areas of PEN2 was about 5 m. Gas concentrations were continuously acquired according to a measurement cycle. The measurement cycle (i.e., composed of the numerical sequence of the SLs) was optimised with the aim of reducing bias due to the detection of very different concentrations (i.e., high and low concentrations) between two adjacent SLs, as suggested by Rom and Zhang [13].

During the data collection, an INNOVA device was used to perform three repetitions for each SL before switching to the next SL. The detection limits, declared by the manufacturer, are as follows: 0.2 ppm for NH_3_, 0.4 ppm for CH_4_, and 1.5 ppm for CO_2_. Moreover, INNOVA required about 1 min 15 s for each repetition, about 4 min for each SL, and less than 1 h to conclude a measurement cycle.

### 2.3. Processing Dataset for the Assessment of the Variability of Gas Concentrations in Different Groups of SLs

Collected data were utilised to create a dataset with two main parameters for each gas: the first parameter was the mean value of gas concentration $\bar{x}$ obtained by averaging concentration values measured in the three repetitions for SL; the second was the variability $s_{x,n}$, expressed as a percentage, as the ratio between standard deviation $s_{x}$ and the mean value of the three repetitions ($\bar{x}$) in the SL considered.

The variability $s_{x}$ (ppm) was computed by using the equation of standard deviation (SD) applied to the three repetitions for SL:

$$s_{x}=\sqrt{\frac{\sum_{i=1}^{n}{\left(x_{i}-\;\bar{x}\right)}^{2}}{n-1}}\equiv \;SD$$

where *n* is the number of data points (i.e., 3), $x_{i}$ is the *i*-th value of the measured data, and $\bar{x}$ is the mean value of *x_i_*. The quantification of the variability $s_{x,n}$ (%) was carried out by normalising the values of the standard deviation *s_x_* by the mean value of gas concentration:

$$s_{x,n}=\frac{s_{x}\;}{\;\bar{x}}\times 100$$

The rows of the dataset include the following elements: gas type (i.e., NH_3_, CH_4_, CO_2_); SLs group (i.e., central, perimeter, corner); $\bar{x}$; $s_{x}$; and $s_{x,n}$.

To compute the variables $\bar{x}$ and $s_{x,n}$ of this first dataset, all the outliers of the mean values of the gas concentrations were computed for both the observation period and the daily trend. Each mean value that was classified as an outlier for both the observation period and the daily trend was excluded from the dataset.

The resulting dataset, named Dataset 1 hereafter, was organized for each gas by randomly selecting the same number of measurements for each group of SLs (i.e., central SLs, perimeter SLs, and corner SLs).

### 2.4. Processing Dataset for the Assessment of the Variability of Gas Concentrations for a Different Number of SLs

Based on the data collected, two new datasets were produced for each gas: the first referred to the central SLs and the second to the perimeter SLs. For each dataset, the gas concentration in the specific area of the barn was computed by considering different replicates of SLs in space (i.e., the mean values of the $\bar{x}$ at different SLs).

Data acquired at four different SLs were used to compute the combination of measures in order to calculate the mean values of gas concentrations acquired at 1, 2, or 3 SLs. A combination determines the number of possible arrangements in a collection of items where the order of the selection is not relevant. In detail, the number of SLs considered was modified from one to three sampling points, but it was not considered how the sampling distribution was organised in the barn.

Therefore, gas concentrations in the central/perimeter area were subdivided into three groups based on the combinations (from 1 to 3) of SLs utilised for the estimation.

In the first group, gas concentration was computed from the measurement $\bar{x}$ recorded in each one of the four SLs in central/perimetral area. In the second group, gas concentration was calculated as the mean value between gas concentrations at two different combinations of SLs (SL03-SL04, SL03-SL05, SL03-SL06, SL04-SL05, SL04-SL06, and SL05-06 in central area; SL08-SL09, SL08-SL10, SL08-SL11, SL09-SL10, SL09-SL11, and SL10-11 in the perimeter area). The third group was composed of gas concentrations computed as the mean value of three different combinations of SLs (SL03-SL04-SL05, SL03-SL04-SL06, SL04-SL05-SL06, and SL03-SL05-SL06 in the central area and SL08-SL09-SL10, SL08-SL09-SL11, SL09-SL10-SL11, and SL08-SL10-SL11 in the perimeter area).

The reference measurement of the gas concentration (benchmark) was determined by the mean values of gas concentrations in all four central SLs (SL03, SL04, SL05, and SL06) or in all four perimeter SLs (SL08, SL09, SL10, and SL11).

In order to study the variability $\varepsilon_{i}\left(\%\right)$ in the estimation of gas concentration due to the number of SLs, the following relation was applied:

$$\varepsilon_{i}=\frac{\left|GC_{rif}-GC_{i}\right|}{GC_{rif}}\times 100$$

where $GC_{rif}$ (ppm) was the reference measurement of gas concentration and $GC_{i}$ (ppm) was the gas concentration value determined by using the *i*-th combination of SLs.

The final dataset, named Dataset 2, was obtained by randomly selecting the same number of measurements for each group; it was used with the aim to determine the influence of replicates of SLs in space on the accuracy of measurements.

### 2.5. Processing Dataset for the Assessment of the Variability of Gas Concentrations for SLs Located at a Different Distance

This dataset, named Dataset 3 hereafter, is based on the variability of gas concentrations computed in Dataset 2. In detail, the variability of gas concentrations computed in the central SLs was subdivided into two groups. The first group considered SLs located at a 5 m distance (i.e., SL03-SL04, SL04-SL05, SL05-SL06), whereas the second group was composed of SLs located 10 m from each other (i.e., SL03-SL05, SL04-SL06). The resulting Dataset 3 was organised for each gas by randomly selecting the same number of measurements for each group of SLs (i.e., 5 m, or 10 m).

### 2.6. Data Assessment and Statistical Analyses

Data were analysed using the software Microsoft Excel^®^ and Minitab^®^. Data analyses were carried out in order to assess the variability of data within the plan distribution of SLs. Based on Datasets 1, 2, and 3, statistical analyses were carried out by applying one-way analysis of variance (ANOVA). The one-way ANOVA statistically tested the differences between different groups of data, described in the following subsections. In each post hoc analysis, the mean values were separated by Tukey’s honestly significant difference at *p* < 0.05.

Since the sampling strategies required analyses of the number and localisation of SLs, the following analyses were carried out:

1. *Localisation of SLs.* In the first analysis, the plan distribution of SLs was assessed by using data from Dataset 1. First, $\bar{x}$ was computed for each gas. The statistical differences were assessed for the three groups of SLs (i.e., central SLs, perimeter SLs, and corner SLs). Finally, $s_{x,n}$ was computed for each gas based on the above-mentioned groups of SLs.
2. *Number of the replicates in space.* In this analysis, different SLs in pen 2 were assessed as replicates of SLs in space. Starting from Dataset 2, the $\varepsilon_{i}$ was investigated by using three groups of data with one, two, or three replicates of SLs during the measurement procedures.
3. *Distance of the SLs.* The statistical test was carried out to assess significant differences for the two groups of SLs with a 5 m and 10 m distance between two SLs.

## 3. Results and Discussion

The statistical analyses carried out on the variability $s_{x,n}$ for the three groups of SLs corroborated the results of the previous analyses found in the study by D’Urso et al. [12]. The variability $s_{x,n}$ presented a significant difference (*p* < 0.001) for NH_3_, CO_2_, and CH_4_ with the changing of the location in the barn. As shown in Table 1, the corner SLs had the highest variability for all the gases analysed (i.e., NH_3_, CH_4,_ and CO_2_). These outcomes were in line with those by König et al. [14] that found a better precision (smaller random error) for all SLs near the centre of the barn than those at the corner.

The lowest mean value of $s_{x,n}$ for NH_3_ was found at the central SLs confirming that the localisation of the SLs is suitable for this gas. Conversely, the higher $s_{x,n}$ for CH_4_ could suggest that it was subjected to dispersion and flushing.

The gas with the lowest variability was CO_2_ compared to NH_3_ and CH_4_. Based on this result, the CO_2_ is a suitable gas to be used as a tracer gas in these open structures in order to estimate emissions [23].

For each gas, the highest gas variability was found in the corner SLs. This could be attributed to the high variability of ventilation, and consequently, it is advisable that measurement procedures should neglect corner SLs. Based on these outcomes, outdoor and indoor SLs should be located with the objective to increase the number of data used for the ventilation rate [12], as well as to reduce errors due to spatial variation [6,15]. The VERA protocol [15] requires at least two meters distance between the SL and the side wall or the outlet opening. The results for this open barn typology corroborate the measurement strategy of VERA protocol, which suggests not performing measurements near the openings. However, the barn facility under study did not allow us to locate SLs in an intermediate position between the central SLs and the perimeter SLs.

Several research studies have shown that the daily gas production was not uniform in the barn and varied at different SLs [11,14]. Results of the Tukey test on the number of SLs (Table 2) showed that it is difficult to find one individual representative SL, as found by König et al. [14], yet it shows that it is better to consider three SLs because the obtained variability is significantly lower than if considering one or two replicates in space. Since the concentration measured at the SLs should be representative of the average concentration in the building [3], the number of SLs utilised for determining the mean value of gas concentration in the barn had a significant influence for NH_3_ (*p* < 0.001), CO_2_ (*p* < 0.001), and CH_4_ (*p* < 0.001) in both the central zone and the perimeter one. Specifically, by increasing the number of SLs in the computation of the mean value, the variability decreases to about 6% for NH_3_, 2% for CO_2_, and 8% for CH_4_ in the central zone, whereas it reaches 10% for NH_3_, 2% for CO_2_, and 16% for CH_4_ in the perimeter zone. These results are in line with those reported in other studies [6,14]. In fact, the measurements of gas concentrations should be carried out simultaneously for multiple locations to improve the representativeness of the measurement values. Moreover, the number of replicates in space can contribute to obtaining a representative mean value of gas concentration in the barn, which is highly useful for the estimation of gas emissions [11,19]. Among the main influencing factors, climatic parameters as well as barn management can increase the variability of air exchange rate and emissions [10,24,25,26]. Increasing replicates in space up to three SLs could reduce the local effect of these variables. Specifically, the variability decreases under 10% in the central zone and under 16% in the perimeter one. However, two replicates for NH_3_ produced a variability of about 10% for the measurement of gas concentrations in the central pen. Consequently, despite the variability of the 10% gas concentrations, it was possible to use two SLs in that area of the barn for NH_3_ monitoring. Since the considered INNOVA system monitored up to 12 SLs in the barn, the results of this study about plan distribution of SLs will allow the optimisation of SL design in order to monitor a wider surface in the barn. With regard to CH_4_, the higher variability could be related to the vertical position of the SLs. In fact, the height of 0.2 m from the floor is low in relation to the main source of the gas production, i.e., the animals. Improving our knowledge of the vertical location of SLs could be useful to further analyse the CH_4_ variability.

Since the number of SLs significantly affected the variability of the data, the influence of the distance between two SLs was further investigated in the central zone due to the lower influence of external variables [12]. Specifically, it was found that the variability in NH_3_ concentration computed at two SLs at a 5 m distance was significantly higher than those computed in two SLs located at 10 m (Table 3). This could be related to the uneven gas distribution in the barn. In fact, the variability of gas concentration obtained by the mean value of two SLs at a 5 m distance was higher than the variability obtained by the mean value of two SLs located at a 10 m distance. This result could be related to the effect of the gas dilution and flushing along the longitudinal axis of the barn, as found in previous studies [12,27]. In fact, the mean NH_3_ concentrations acquired in two SLs at a 5 m distance mainly overestimate the average gas concentration at the four locations (i.e., the benchmark) more than the mean of the NH_3_ concentration of the two SLs at a 10 m distance. In detail, the position of the SLs at a 5 m distance covers a smaller area of the barn compared to SLs at a 10 m distance. Therefore, the effect of the dilution and flushing is more evident when the mean value is computed on NH_3_ concentrations acquired at SLs located at a 10 m distance. Consequently, the distance of 5 m is less representative compared to those of 10 m for NH_3_. It is not possible to confirm the same for CO_2_ and CH_4_ because the statistical test did not show a significant difference. Since this result could be ascribed to the vertical position of SLs, further investigations could be carried out with a different distribution of SLs.

## 4. Conclusions

In this study, the concentrations of NH_3_, CO_2_, and CH_4_ were measured in an open dairy barn during late spring. The results of this work quantified the variability in gas concentrations related to the sampling strategy in the horizontal distribution and number of sampling locations (SLs). By using statistical analyses, it was found that the variability in the gas distribution was significantly influenced (*p* < 0.001) by different positions of SLs, the number of SLs, and the distance between two consecutive SLs in the barn environment.

Within the context of precision livestock farming, further studies could be aimed at analysing the optimum vertical position of SLs, the minimum distance of SLs from the perimeter, and the influence of climatic factors or barn management (e.g., operation of the fans, activation of cooling systems, number of milkings, and cleaning of the floor) in the uncertainty of measurements. Moreover, further research studies could investigate whether the requisites defined in guidelines for naturally ventilated barns (i.e., VERA Protocol) could also be applied in different open barn typologies, characterised by the absence of walls, in the Mediterranean area.

Based on the improved knowledge of measuring gas concentrations, the following step will involve the evaluation of the impact of different strategies for measuring gas concentration on the emission estimation for open dairy houses.

## Figures and Tables

**Table 1:** Gas variability *s_x,n_* in three different groups of sampling locations for each gas, and outcomes of post hoc analysis (Tukey test).

Gas	Zone	$\mathrm{Mean}\;s_{x,n}\;(\%)$	Grouping		
NH_3_	Corner SLs	9.96	A		
	Perimeter SLs	8.21		B	
	Central SLs	6.84			C
CO_2_	Corner SLs	4.87	A		
	Central SLs	4.35		B	
	Perimeter SLs	2.43			C
CH_4_	Corner SLs	31.03	A		
	Central SLs	20.14		B	
	Perimeter SLs	7.49			C

Rows with a different letter (A, B, C) are significantly different.

**Table 2:** Results of the variability $\varepsilon_{i}$ in relation to the number of sampling locations considered, in the central and perimeter zones, and outcomes of post hoc analysis (Tukey test).

		Central Zone				Perimeter Zone			
Gas	Number of SLs	Mean ε(%)	Grouping			Mean ε(%)	Grouping		
NH_3_	1	17.16	A			28.90	A		
	2	10.08		B		17.17		B	
	3	5.72			C	9.63			C
CO_2_	1	5.94	A			4.87	A		
	2	3.43		B		2.86		B	
	3	1.98			C	1.63			C
CH_4_	1	24.07	A			46.83	A		
	2	13.89		B		26.68		B	
	3	8.02			C	15.61			C

Rows with a different letter (A, B, C) are significantly different.

**Table 3:** Results of the variability $\varepsilon_{i}$ in relation to the distance of the two sampling locations considered, and outcomes of post hoc analysis (Tukey test).

Gas	Distance	Mean $s_{x,n}\;(\%)$	Grouping		
NH_3_	5 m	11.26	A		
	10 m	8.4		B	
CO_2_	5 m	3.38	*p* = 0.23		
	10 m	3.18			
CH_4_	5 m	14.37	*p* = 0.09		
	10 m	13.31			

Rows with a different letter (A, B) are significantly different.

## Data Availability

Data available on request due to privacy restrictions.
